# Glial-Mediated Inflammation Underlying Parkinsonism

**DOI:** 10.1155/2013/357805

**Published:** 2013-07-11

**Authors:** Carlos Barcia

**Affiliations:** Department of Biochemistry and Molecular Biology, Institute of Neuroscience & School of Medicine, Universitat Autònoma de Barcelona, Campus de Bellaterra, Cerdanyola del Vallès, 08193 Barcelona, Spain

## Abstract

The interest in studying neuroimmune interactions is increasing in the scientific community, and for many researchers, immunity is becoming a crucial factor in the understanding of the physiology of the normal brain as well as the biology underlying neurodegenerative diseases. Mounting data over the last two decades point toward immune and inflammatory alterations as important mediators of the progressive dopaminergic degeneration in Parkinson's disease. The purpose of this review is to address, under a historical perspective, as well as in the light of recent reports, the glial-mediated inflammatory and immune responses that occur in Parkinsonism. In line with this, this review also evaluates and highlights available anti-inflammatory drugs and putative targets for Parkinson's disease therapy for the near future.

## 1. Introduction

After many decades of research, the cause of idiopathic Parkinson's disease (PD) remains unknown. A number of hypotheses have been put forward to explain the origin of the disease. However, the understanding of the mechanisms underlying PD remains inconclusive. The trigger of dopaminergic degeneration seems to be multifactorial and, therefore, affected by both endogenous and environmental elements. In the light of recent epidemiological, genetic, and experimental studies, inflammation and immune responses are considered as important mediators of dopaminergic degeneration. Large population studies have come to conclude that individuals taking nonsteroidal anti-inflammatory drugs (NSAIDs) have less risk of suffering idiopathic PD, which suggest that anti-inflammatory drugs may be a promising disease-modifying treatment for Parkinsonian patients [1–4]. Important genetic studies have shown an increase of polymorphisms of the human leukocyte antigen (HLA)-DR type gene in sporadic PD, indicating an immune/inflammatory-related component of the disease [5, 6]. Despite the extended basic research performed in experimental models of PD and the positive outcome of a wide range of tested anti- inflammatory drugs, the translational aspect toward a neuroimmune-modifying therapy in PD has been rather slow. In recent years, some pharmacological companies have taken steps towards the development of therapeutic programs. New trial phases have recently been started to implement anti-inflammatory treatments for the near future. There are a number of clinical trials, essentially focused on monitoring the evolution of the inflammatory response in the brain of PD patients *in vivo*, using potential imaging biomarkers in the course of dopaminergic degeneration. [123-I] CLINDE, [18F] FEPPA, and [(11)C] PBR28 are some of the compounds that are being evaluated in Europe and North America for their capacity to detect neuroinflammation in Parkinsonian patients by single-photon emission computed tomography (SPECT) (source: NIH website, http://www.clinicaltrials.gov/). The outcome of these trials will provide crucial data to test and monitor the progression of anti-inflammatory treatments for PD in the future and will help to define the timely therapeutic window to avert, or at least decelerate, inflammatory-mediated dopaminergic degeneration. In the following sections of this review, the inflammatory and immune responses, previously described in Parkinsonism, are evaluated in a historical perspective. Then, considering the recent advances achieved in PD patients and animal models of PD, the main aspects and mechanisms of glial-mediated inflammation during dopaminergic degeneration are reassessed, suggesting putative and inflammatory drugs for therapeutic purposes.

## 2. Historical Perspectives on Inflammatory Response in Parkinsonism

### 2.1. Postencephalitic Parkinsonism: An Immune-Mediated Parkinsonian Syndrome

Since the description of the encephalitis lethargica by von Economo in 1917, the idea that inflammatory responses or immune-mediated events might contribute to the degeneration of dopaminergic neurons had been suggested [7]. The patients affected by von Economo's encephalitis displayed clinical Parkinsonian signs that overlapped with the idiopathic PD syndrome and showed degeneration in basal ganglia areas and midbrain neurons of the substantia nigra (SN) [8]. The necrosis of the SN could be either unilateral or bilateral and showed a widespread gliosis [9] with prominent glial scars in the SN [10]. In the late 60s and early 70s, new isolated cases of encephalitis with Parkinsonian symptoms also confirmed the inflammatory necrosis of the SN [8, 11], suggesting that specific inflammatory responses might have neurological effects with Parkinsonian appearance.

 The cause of postencephalitic Parkinsonism remains uncertain. It is thought that a viral infection could be the trigger of the disease. Interestingly, in the study of recent cases, the fact that specific variants of influenza virus, like H5N1, may cause encephalitis, together with the experimental verification that it can be transferred from birds to mammals, supports the hypothesis that H5N1 epidemic infections may have Parkinson-like neurological consequences [12]. In line with this, a recent review highlights that among all the influenza epidemics that occurred in the 20th century, only von Economo's encephalitis had a well-described Parkinson-like syndrome [13]. Furthermore, H5N1 influenza virus is able to enter the brain and induce Parkinsonism in mammals, which makes this type of virus the most plausible cause for the 1917 pandemic [14]. 

These facts and historical evidence suggest a clear parallelism between postencephalitic Parkinsonism and idiopathic PD and sustain the hypothesis of a possible common factor for both Parkinsonian syndromes. Postencephalitic Parkinsonism was initially treated with anti-inflammatory drugs, usually corticoids, because an infectious origin was assumed. However, the clinical and histological coincidences with PD suggested the use of L-DOPA as a treatment for Parkinsonian encephalitis. Initially, patients had an encouraging and surprising positive response to L-DOPA treatment, but unfortunately the response was reversible not durable and induced severe side effects [15].

After von Economo's epidemic, the link between Parkinsonism and immunity has been further analyzed, and has led to hypothesize that infectious or immune-related factors may critically affect PD [7, 16]. Since the origin of PD is unknown, the comparison between both syndromes is still intriguing. The fact that a well-known inflammatory or immune response is able to induce a particular Parkinsonism in encephalitis lethargica suggests that idiopathic PD could as well be caused, or at least aided, by an underlying, yet poorly understood, immune or inflammatory response.

### 2.2. First Descriptions of Glial-Mediated Inflammatory Responses in PD and MPTP-Induced Parkinsonism

In 1988, McGeer and coworkers described for the first time that the areas of dopaminergic degeneration of brains from patients who died with PD showed clear signs of neuroinflammation, characterized by the activation of microglial cells [17]. Importantly, the activation of microglia was detected through the increase of the HLA-DR expression, suggesting active nerve degeneration. The impact of this publication at that time was discrete, since it seemed obvious that local neuro-inflammation in the neighboring glia was a causative effect of the neuronal degeneration. However, McGeer's publication became years later one of the most prominent breakthroughs for the understanding of the inflammatory responses in PD.

In 1999, the publication of the he first *postmortem* analysis performed in three cases of the so-called *Frozen Addicts,* described by Dr. Langston, transformed the scenery of this field of research [18]. In 1982, in Santa Clara, California, a group of young people was diagnosed with a severe Parkinsonian syndrome, showing almost identical clinical signs to idiopathic PD. All of them were drug addicts who received heroin from a common provider. Then, Dr. Langston and his team, in collaboration with the National Institute of Health, determined that the heroin that they consumed was contaminated with a neurotoxin called 1-methyl-4-phenyl-1,2,3,6-tetrahydropyridine (MPTP) that injected intravenous in primates causing a permanent Parkinsonian syndrome [19, 20]. Three of these patients died at the end of the 90s, and the first *postmortem* studies revealed that the SN cells were depleted, as what occurred in PD patients [18]. The areas of dopaminergic degeneration showed active microglia, expressing high levels of HLA-DR, identical to McGeer's observations described years before in PD brains [18]. This data demonstrated that a still active nerve degeneration was ongoing many years after the neurotoxic insult, suggesting that microglial cells may initiate a neuroinflammatory cycle in the areas of degeneration that contribute to the neuronal death. Importantly, in these particular cases, a single set of injections of MPTP, taking place almost 15 years before, were able to initiate a persistent inflammatory response affecting the conditions of remnant dopaminergic neurons for decades. This phenomenon was mimicked in monkeys, and microglial activation could be observed in the SN years after the neurotoxin insult [21] even without L-DOPA treatment [22], which supported even more strongly that solely the initial MPTP insult was able to originate a persistent neuroinflammation in dopaminergic areas. Therefore, the question that had to be answered, and still has, is whether this glial-mediated inflammatory response is able to induce or influence neuronal degeneration.

## 3. Glial-Mediated Inflammation in Parkinsonism

### 3.1. Role of Microglial Cells in the Proinflammatory Environment in Parkinsonism

The study of the inflammatory response in PD has been essentially focused on the study of microglia in the neurodegenerative process [23–25]. Despite the increasing data and publications over the last two decades, microglia's role in PD is still unclear and not fully understood. The reason why microglial cells remain activated during years in Parkinsonian individuals, such in PD- and MPTP-intoxicated patients [17, 18], is still a difficult fact to evaluate and understand. The expression of HLA-DR in humans is usually associated with active neuronal degeneration, but there are reasonable doubts on whether microglial activation is a cause or a mere consequence of the neuronal death. In humans and nonhuman primates, the exploration of microglial activation at different time points entails serious technical limitations. It becomes really challenging to assess which phenomenon occurs first and whether long-term neuro-inflammation is able to induce new dopaminergic degeneration *in vivo* or not [21, 22]. In patients with PD, studies performed with position emission tomography with a radiotracer for activated microglia [(11)C](R)-PK11195 and a dopamine transporter marker [(11)C]CFC show interesting and clarifying results. Microglial activation can be detected in patients in the nigrostriatal pathway *in vivo* through the radiotracer [(11)C](R)-PK11195, which appears increased in PD compared with healthy subjects. Importantly, patients with early PD show a significant correlation between microglial activation [(11)C](R)-PK11195 and dopaminergic terminal loss [(11)C]CFC, which indicates a direct effect of inflammation on neuronal degeneration [26]. However, in long-term Parkinsonian patients, microglial activation measured by [(11)C](R)-PK11195 remains persistently increased in stable levels during years even when the dopaminergic loss progresses over time [27]. Coherently with these results, *postmortem* studies in monkeys show that either acute or chronic protocols of MPTP administration are able to induce similar levels of microglial-mediated inflammatory responses [28].

**Figure 1:**
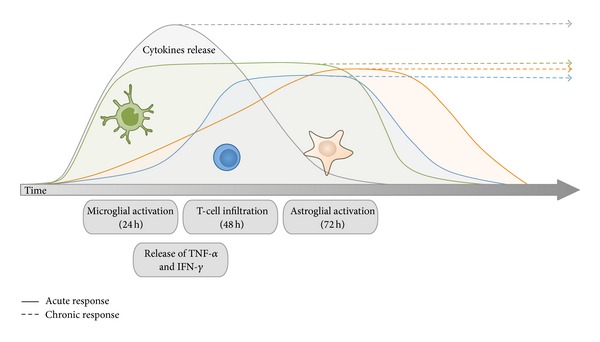
Timing of cytokine release, glial activation, and T-cell infiltration in the inflammatory response in Parkinsonism. Within 24 h, cytokine release and microglial activation can be clearly observed. According to the protocol of dopaminergic cell death induction, cytokine release and microglial activation can be resolved around 72 h. Maximum peak of T-cell infiltration can be achieved at 48 h after dopaminergic insult. Astroglial activation can be seen at 24 h but reaches the maximum peak at 72 h. Depending on the protocol of dopaminergic cell death induction used, the inflammatory response may be resolved within 72 h.

This evidence indicates that in humans and non-humans primates, microglial activation gets rapidly activated in the first stages of dopaminergic degeneration and then maintained active during years once a particular threshold of activation is reached. Therefore, glial-mediated inflammation in Parkinsonism in primates seems to be critical in the early phases of dopaminergic degeneration and indicates that this period may be relevant as a timely therapeutic window. By contrast, in Parkinsonian mice, microglial activation is transient and goes back to basal levels when the dopaminergic degenerative process is resolved (Figure 1) [29, 30]. Very little is known about why glial cells perform differently between species but it is clear that rodents and primates have a different regulatory mechanism driving glial responses after dopaminergic insult. The investigation of these species-dependent differences may represent one of the key pieces to understand the neuropathological puzzle that links glial-mediated inflammation with neuronal degeneration. 

**Figure 2:**
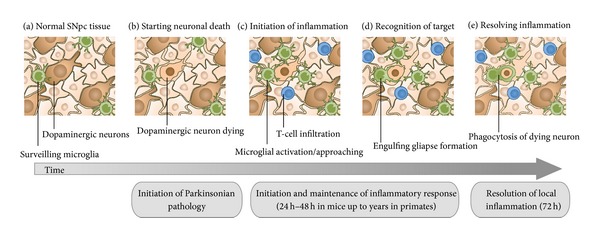
Local inflammatory response to dopaminergic neuronal death. (a) In normal SNpc tissue, microglia screen the brain parenchyma for changes. (b) In Parkinsonism, when dopaminergic neurons start dying, a number of signals, such as ATP or Ca^2+^, are released into the parenchyma. (c) These signals induce the inflammation, characterized by the enrolment of neighbor microglial cells and the recruitment of blood cells, such as T-cell lymphocytes or monocytes. (d) Microglial cells approach and contact damaged neurons. (d) Active microglial cells establish engulfing gliapses with degenerating neurons. (e) Microglial cells engulf and phagocytose degenerating neurons to resolve the local inflammation. The inflammatory response starts 24 h after the dopaminergic insult and is maintained during days until it is resolved. In mice, it could be resolved within 72 h, while in primates it could continue for years.

**Figure 3:**
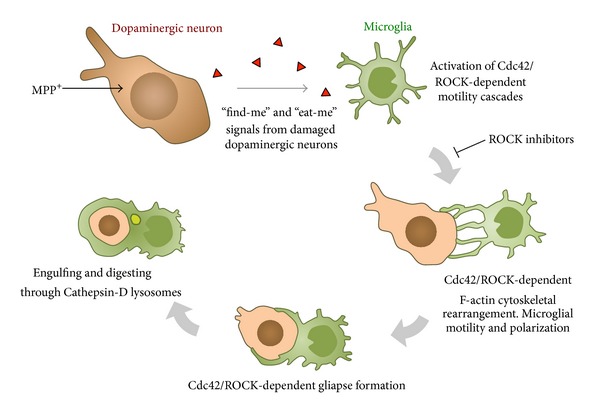
Graphical diagram of the motility and polarization of microglial cells in dopaminergic degeneration. Dopaminergic neuronal death generates the release of products, such as ATP or Ca^2+^, which activates the surrounding microglia. These gradients are able to attract microglia toward neurons (find-me signal). Cdc42/ROCK-dependent signaling controls the motility and polarization of microglia, which can be blocked by specific ROCK inhibitors. The motility of microglia starts with the polarization of microglial processes and then the apposition of the microglial cell body toward the neuron, forming an engulfing gliapse. In the engulfing process, dopaminergic neuron nucleus starts to show chromatin condensation and displays a pyknotic appearance. Cathepsin-D lysosomes digest the content of the phagosome (modified from [42]).

The triggering of microglial activation is mediated by several factors. In basal conditions, microglial cells are in constant surveillance in the brain parenchyma and are susceptible to undergo important morphological arrangements according to the changes of the microenvironment [31, 32]. The initial steps of the neuronal degeneration are associated with the release of a number of signals that induce the microglial activation and polarization toward the damaged neurons. It is known that P2Y receptors are very important players in the motility and polarization of microglial cells [33, 34]. Degenerating neurons release or leak ATP, which activates microglial P2Y receptors, attracting microglia toward the ATP gradient [33–35]. A recent report also demonstrates that degenerating neurons signal particular calcium waves, attracting neighboring microglial cells into the area of local damage [36]. These signals recruit microglial cells, which are able to move their branches and their cell bodies toward the area of degeneration [33]. The final purpose of this motility is to restore the tissue and remove debris from the areas of degeneration (Figure 2), but the details of how this process takes place in adult mammals in the neurodegenerative process are not fully understood. It is thought that microglial cells move to engulf particles as a key function of the immune response in the brain. Previous reports have shown that microglial cells are able to phagocyte fluorescent microspheres, opsonized beads, or fluorescently labeled *β*-amyloid, which addresses the phagocytic properties of microglia [37–39]. Experiments performed in zebrafish embryo have demonstrated that microglia phagocytose neurons in brain development [40]. On the other hand, in adults, apoptotic newborn cells are phagocytosed by microglial branches forming ball-and-chain structures, a crucial phenomenon for the homeostasis of the brain parenchyma [41]. Although it is assumed that microglia may phagocyte neurons in CNS diseases, how the engulfing process takes place in neurodegeneration is still under investigation. Recently, we have reported that microglial cells phagocytose entire dopaminergic neurons in a one-to-one ratio in a mouse model of PD. This process involves a complex machinery where microglia arrange their F-actin cytoskeleton forming a structure, named gliapse, in which the entire microglial cell body is closely apposed to the damaged neuron, polarizing its filopodia toward the neuronal cell body and placing the organelles toward the cell-to-cell interface (Figure 2) [42]. Microglial cells, following “find-me” and “eat-me” signals released from degenerating neurons, activate Rho-kinase- (ROCK-) and Cdc42-dependent motility cascades, which are crucial in the cytoskeletal rearrangement (Figure 3). Microglial motility and polarization end up in the intimate apposition to the degenerating neuron, engulfing and digesting the dying neuronal body [42]. These lines of evidence give new details and insights of how microglial cells contribute to the dopaminergic degeneration. Further studies in experimental models of PD and the comparison with the human disease will be critical to better understand the particular role of microglia in this pathological scenario and find new therapeutic approaches to arrest microglial cells and to avert proinflammatory environment of PD.

### 3.2. Role of Astrocytes in the Proinflammatory Environment in Parkinsonism

Besides the prominent functions played by microglial cells, astrocytes also participate actively in the neuroinflammatory response [43]. Astrocytic reaction has generally been considered as an essential event in forming the so-called glial scar [44, 45], however, in the light of more recent data, it is well known that astrocytes play a more complex role in the neurodegenerative and restorative process [44, 46–48]. Astroglial cells become reactive in many neurodegenerative alterations such as PD [49]. The areas of neurodegeneration of PD patients show high expression of glial fibrillary acidic protein (GFAP) [17, 50, 51], which is a compound of the astrocytic cytoskeleton that indicates reactivity of astrocytes. Astrogliosis is seen in other forms of Parkinsonism, such as postencephalitic Parkinsonism [52] and MPTP-induced Parkinsonism in intoxicated humans [18] or non-human primates [22]. As microglial cells, astrocytes become reactive after the dopaminergic insult. In mice, the astrogliosis is transient, while in primates it persists during months or years (Figure 1). The presence of astroglial cells with reactive phenotype, such as the increase of GFAP, is considered part of a neuroprotective process [46, 53]. In the case of the dopaminergic pathway in Parkinsonism, the most vulnerable regions of the mesencephalon, specifically the subregions of the SNpc, show very low density of astroglial cells compared with less vulnerable dopaminergic areas of the mesencephalon, which suggests that the endogenous presence of astroglia represents a factor for neuroprotection [54]. However, the mechanisms driving this protective role in Parkinsonian degeneration are still unclear. Astrocytes are able to release many factors that may contribute to the restoration of the degenerated tissue [55]. Glial-derived neurotrophic factor (GDNF), among others, is a good example. GDNF is produced and released by astrocytes [56] and it has a beneficial effect in experimental models of Parkinsonism [57–59]. Unfortunately, GDNF therapeutic benefit for PD patients has been controversial and not conclusive [58, 60]. Other factors, such as astrocytic brain-derived neurotrophic factor (BDNF) [61, 62], neurotrophin-3 (NT-3) [62], or mesencephalic astrocyte-derived neurotrophic factor (MANF) [63] are also involved in the neuroprotective and restorative role in brain damage.

On the other hand, astrocytes also contribute to the inflammatory environment and facilitate the persistency of the neurodegenerative process through the production and release of proinflammatory cytokines [64]. Astrocytes are able to produce and release a number of cytokines [65–67] under the stimulation by different inflammatory-dependent factors [65, 66, 68], having a clear impact in the neurodegenerative processes. This topic is extensively reviewed in Section 4 of the present paper. However, astrocytic-derived cytokines have other unsuspected implications such as the control of neurotransmission at synaptic level, which goes beyond the glial-mediated proinflammatory reaction [69, 70].

The particular anatomical location of astrocytes, near endothelial cells, is also important for the maintenance of the homeostasis and the regulation of the inflammatory environment. Specifically, the secretion of specific cytokines and chemokines at the verge of blood vessels is a crucial phenomenon in regulating the extravasation of blood cells at the areas of degeneration [51, 71, 72]. Astrocytes are responsible of the production and release of some chemokines, such as CCL2, CCL3, and CCL5, which are fundamental for the infiltration of macrophages and lymphocytes in the brain parenchyma. This anatomical location is also important to eliminate debris produced in the brain parenchyma through the draining fluids. A recent report has shown that astrocyte participates actively in the clearance of interstitial solutes (as amyloid-*β*) through the aquaporin-4 channels, sinking the content to the CSF [73]. This suggests that astrocytes may also play important roles in other degenerative processes such as PD. Unraveling the multifaceted functions of astrocytes in neurodegenerative diseases, and specifically in dopaminergic degeneration, will be a crucial aspect to be pursued in the future research.

### 3.3. Role of Oligodendroglia in the Proinflammatory Response in Parkinsonism

The role of oligodendroglia in the Parkinsonian pathology remains unclear and it has been mostly ignored. Few studies have been published reporting oligodendroglial alterations in Parkinsonism [74]. One of the limitations to study this phenomenon lies in the fact that nigrostriatal dopaminergic fibers are poorly myelinated. Besides, oligodendrocytes are very shifting and complex cells; thus, their alterations due to neurodegeneration are difficult to address in the adult brain. Oligodendrocyte phenotype and protein expression change during axon re-myelination [75], and it is reasonable to think that similar changes may occur in oligodendrocytes after neuronal degeneration. In response to demyelinating injury, oligodendrocyte precursor cells undergo changes in morphology and upregulate several transcription factor genes, such as OLIG2, NKX2.2, or MYT1 [75], to initiate the process of remyelination. However, the response of mature oligodendrocytes after axon loss remains scarcely explored.

Very few studies have been published regarding oligodendrocyte reactions after dopaminergic axon loss in PD. The presence of complement-activated oligodendrocytes in the SN of PD patients has been described [76], but its biological significance and the link with the inflammatory response remain unclear. Other groups have described the presence of inclusions of *α*-synuclein in oligodendrocytes of the nigrostriatal pathway of patients with PD [77], which suggests a direct implication of this cell type in the neuropathological disease.

Regarding experimental models of PD, myelinated fibers appear disrupted in MPTP-induced Parkinsonism in mice [78], but little information is given about the state of the wrapping oligodendrocytes around dopaminergic fibers after degeneration. A recent report has shown that mature oligodendrocytes, expressing myelin basic protein (MBP), are overreactive in MPTP models of PD [79]. In Parkinsonian mice, MBP-oligodendrocytes appear increased in numbers and with a reactive phenotype, characterized by a larger cell body size and an increase of the number of ramifications, selectively in the areas of dopaminergic degeneration [79]. In mice, this reaction disappears few days after the neurotoxic insult, and oligodendrocytes go back to their normal morphological state, similar to the microglial and astroglial reactive phenotypical changes [79]. In primates, however, MBP-oligodendrocytes still display reactive phenotype years after the MPTP insult and appear increased in numbers, showing persistent MBP immune reactivity with respect to controls. Importantly, these changes are concomitant to microglial and astroglial reaction, suggesting an inflammatory-related phenomenon [79]. Nevertheless, the mechanisms driving this changes in oligodendrocytes after MPTP insult and its link with the proinflammatory environment are scarcely explored. Due to their fundamental role in restoration of axons, the analysis of oligodendrocytes in PD should be emphasized and further evaluated. Comprehensive disease-modifying therapies must take into account all cell types, including oligodendrocytes, and be considered as possible cellular targets to treat the disease.

## 4. Proinflammatory Cytokines as Crucial Factors of Glial-Mediated Inflammation in Parkinsonism

### 4.1. Increase of Proinflammatory Cytokines in the Glial-Mediated Inflammation in Parkinsonism

Together with the histological findings obtained from *postmortem* studies, the evaluation of peripheral inflammatory markers, such as proinflammatory cytokines in the blood or cerebrospinal fluid (CSF), has been an important analysis in patients with PD. The first proinflammatory cytokine detected in high levels in the blood and the CSF of patients with PD was tumor necrosis factor-*α* (TNF-*α*) [80]. This finding was relevant because (1) it confirmed that the inflammatory response, taking place in PD patients, goes beyond the brain parenchyma, and (2) because TNF-*α* is a cytokine able to induce cell death through the activation of TNF-*α* receptors (TNF-*α*R). The binding of TNF-*α*R by TNF-*α* stimulates a signaling cascade that activates proapoptotic domains inducing neuronal death [81, 82]. TNF-*α*R are present on the membrane of human dopaminergic neurons, indicating that a TNF-*α*-dependent proinflammatory environment could directly affect the apoptotic signal of vulnerable neurons within the SN of PD patients [83].

In PD experimental models, mice lacking TNF-*α* or TNF-*α*R are less susceptible to MPTP-induced neuro-degeneration [84, 85]. Conversely, the long-term and artificially induced expression of TNF-*α* exacerbates dopaminergic degeneration, together with the stimulation of a sustained inflammatory response in the brain [86]. In Parkinsonian primates, unlike other cytokines, TNF-*α* plays a central role in the long-term inflammatory potentiation of Parkinsonism [87, 88]. However, the question that remains unsolved is whether endogenous circulating TNF-*α* may cause new neuronal degeneration in a self-perpetuated inflammatory environment in primates.

In addition, the proinflammatory cytokine interferon (IFN)-*γ* has also been found increased in plasma of patients with PD, and it has been shown to have an important impact in the inflammatory response involved in dopaminergic degeneration [89]. In experimental models of PD, IFN-*γ* deficient mice are protected against MPTP-induced dopaminergic degeneration and display attenuated local inflammatory response [89]. Since IFN-*γ*R is not present on dopaminergic neuron's membrane, in contrast with TNF-*α*, the role of IFN-*γ* in dopaminergic neuro-degeneration does not affect neurons directly. IFN-*γ* activates bystander glial cells and contributes to the local inflammatory-mediated neuronal degeneration [88, 89]. Recent results obtained from chronic Parkinsonian macaque monkeys show that IFN-*γ* appears elevated in plasma and brain parenchyma, and similar to TNF-*α*, it seems to play a critical role in the long-term maintenance of the inflammatory response in Parkinsonism [88]. Chronic Parkinsonian monkeys maintain elevated amounts of both cytokines, TNF-*α* and IFN-*γ*, during years, and the amounts correlate positively with the degree of Parkinsonism, as well as the level of neuronal degeneration [88]. The importance of the latter results obtained in primates underlies in the fact that they are comparable to the human scenario and could be better extrapolated in terms of therapeutic strategies. Thus, targeting TNF-*α* and IFN-*γ* might be the best approach to diminish Parkinsonian inflammation in a chronic process.

Other proinflammatory cytokines, such as interleukin- (IL-) 1*β*, IL-2, IL-4, and IL-6, were also found elevated in brain, blood, or CSF of PD patients [90–93]. However, their specific function in PD is still poorly understood. Experiments *in vitro* have shown that IL-1*β* and IL-6 are relevant in promoting astroglial reactivation [94]. Some of these cytokines are found elevated after dopaminergic insult in mice and seem to play a critical role in promoting the inflammatory response in acute models of PD in rodents [95–97]. In contrast, in chronic Parkinsonian macaques, no changes were seen in plasma levels of IL-1*β*, IL-16, IL-6, IL-8, and TNF-*β*, which suggest that these cytokines may not play a specific role in the long-term inflammation [88].

Rodents and primates seem to conduct differently regarding the release of cytokines, similar to other proinflammatory parameters such as microglial and astroglial activation. In the case of MPTP-induced Parkinsonian mice, the high levels of circulating cytokines are transient and parallel to the glial inflammatory response observed in the local areas of dopaminergic degeneration in the mouse brain [95–97]. Whereas in primates, circulating cytokines in serum and brain can be detected even years after the initial MPTP insult [88], which indicates that glial activation and the release of cytokines are two phenomena that overlap (Figure 1).

### 4.2. Role of Proinflammatory Cytokines in Glial-Mediated Inflammation and Neuronal Degeneration

The impact of systemic and parenchymal circulation of proinflammatory cytokines on the induction and maintenance of the glial-mediated inflammation* in vivo *has been addressed with different approaches. Elegant experiments performed in models of PD in rodents have shown that circulating cytokines, such as TNF-*α* or IL-1, artificially induced by genetically modified viral vectors, increase the inflammatory response causing a deleterious effect on dopaminergic loss initiated in the SN [98, 99]. The use of the bacteria membrane-derived lipopolysaccharide (LPS) has also been an important tool in order to understand how a non-PD-related-induced inflammation could affect dopaminergic neurons. The administration of LPS in midbrain cultures induces dopaminergic degeneration together with the release of cytokines such as TNF-*α* and IL-1*β* among others [100]. Similarly, the intraparenchymal injection of LPS in rodents also induces a proinflammatory response that is toxic for dopaminergic neurons, which are especially vulnerable to the inflammatory insult [101, 102]. Furthermore, adding LPS in combination with other neurotoxins, such as MPTP, is synergistic and exacerbate both glial activation and neuro-degeneration [103], suggesting that inflammation itself, independent of the source, is deleterious for dopaminergic neurons.

**Figure 4:**
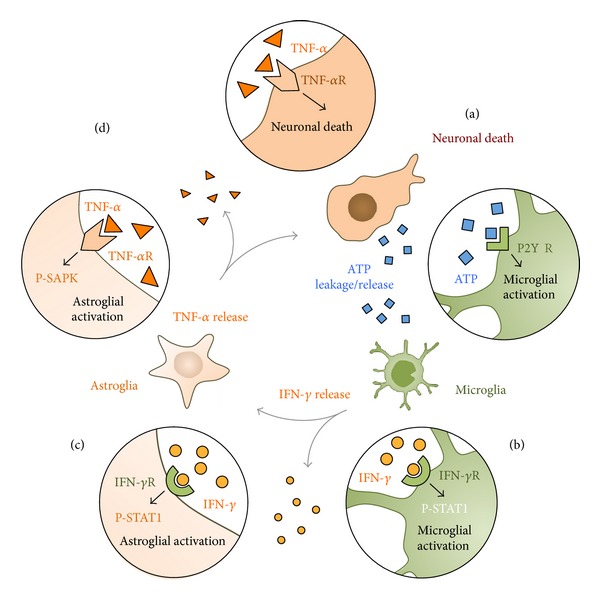
Diagram of the hypothetical vicious cycle of glial activation signaling triggered in Parkinsonism during dopaminergic neuronal loss. (a) Dopaminergic neuronal death induces the release or leakage of ATP, which activates microglial cells through P2YR. (b) Activation of microglia induces the release of IFN-*γ*, which binds IFN-*γ*R in neighbor microglial and astroglial cells initiating the phosphorylation of STAT1. (c) Activation of astrocytes involves the release of TNF-*α*, which may activate astrocytes through the activation of TNF-*α*R by phosphorylating SAPK. (d) Importantly, released TNF-*α* may also activate TNF-*α*R present in dopaminergic neurons, which may induce new neuronal death.

Cytokines function differently according to the specific insults induced in the brain parenchyma [104]. Particularly in PD, the initial cytokine-dependent inflammation may represent an attempt to restore and repair the damage caused in the neurodegenerative process. However, on the other hand, the long-term exposure to increased levels of cytokines could have deleterious consequences for the remnant neurons [54, 105]. It is known that circulating cytokines induce glial activation, which leads to new cytokine release by bystander glial cells. This response creates a vicious cycle where the proinflammatory environment itself may contribute to induce new neuronal degeneration, establishing a chronic process (Figure 4) [88, 106–108]. In summary, the increased levels of certain cytokines, systemically or in the brain parenchyma, could be a self-perpetuating factor of the proinflammatory environment able to contribute and accelerate the neurodegenerative process [109, 110].

From a therapeutic point of view, it is difficult to ascertain which cytokines are the most deleterious for dopaminergic neurons or which ones are able to promote a long-term process. According to previous publications, and with the data obtained so far, TNF-*α* seems to have a direct and prominent role in the Parkinsonian dopaminergic degeneration. However, proinflammatory environments are multifaceted, and the increase of one particular cytokine is accompanied by the stimulation, production, and release of others. Although it is challenging to highlight a single cytokine to block in order to avert inflammation in Parkinsonian degeneration, it is necessary to determine optimal effective therapeutic targets to diminish the proinflammatory environment to develop disease-modifying therapies.

### 4.3. Proinflammatory Cytokines and Glial Cell Crosstalk in Parkinsonism

There is an ongoing debate regarding the cellular source of cytokines in the brain *in vivo*. The most likely candidates for local production and release of proinflammatory cytokines in the brain are the glial cells, mainly microglia and astrocytes [89, 111–113]. In addition, cells coming from lymphoid tissues, the CSF, and the blood, such as monocytes/macrophages or lymphocytes may also participate in the local cytokine production and release in the inflamed brain. 

Studies *in vitro* show that glial cells produce and release proinflammatory cytokines after different stimulus. However, very few studies have been able to demonstrate this *in vivo*. Technically, the detection of cytokines directly in fixed tissue is challenging, and a few commercially available antibodies give a convincing immunostaining in brain tissue. In a recent article of our group, we show with immunofluorescence and detailed confocal analyses the expression of cytokines in glial cells in the SNpc of chronic Parkinsonian macaques [88]. Histologically, we observed that TNF-*α* is expressed fundamentally in reactive astrocytes in the areas of degeneration, while IFN-*γ* appears to be detected only in reactive microglia [88]. The expression of TNF-*α* by astrocytes is well accepted; however, the IFN-*γ* production and release by cells of myeloid origin, such microglia/macrophages, are still controversial [114]. Nevertheless, keeping in mind the controversy, it seems clear that there is a different cellular localization of these two cytokines in chronic Parkinsonism that reflects different contribution for both cell types in the proinflammatory environment. We found that IFN-*γ* receptor is expressed in astrocytes and microglia. Consequently, the downstream cascade of activation of IFN-*γ*R, characterized by the phosphorylation of STAT-1, appears active in astrocytes and microglial cells, suggesting that IFN-*γ* signaling may play an important role in both astroglial and microglial activation [88].

This data was obtained in MPTP-treated primates; thus, it is difficult to ascertain the specific role of each proinflammatory cytokine on the specific activation of glial cell activation because it is a phenomenon that appears overlapped with dopaminergic cell death in the long-term. However, in acute MPTP-induced Parkinsonian models, glial activation can be observed prior to dopaminergic degeneration, which represents an important time window to evaluate the role of cytokines using specific gene deletions with KO animals. In fact, MPTP-treated mice lacking TNF-*α* or IFN-*γ* show important differences regarding glial cell activation before dopaminergic degeneration can be achieved. After MPTP, in the absence of IFN-*γ*, microglial cells get poorly activated, whereas in the absence of TNF-*α*, astroglial cells appear inactive [88]. Thus, IFN-*γ* seems to have a predominant role on microglial activation, while TNF-*α* appears to have a prominent effect on astroglial cell activation. According to these results, cytokine signaling takes place differently in astrocytes and microglia. In this context, the sequential intervention of both cytokines may be very important for the glial activation and its long-term maintenance. As others, we have suggested that astrocytes may work as amplifiers of the inflammatory response initiated by microglial cells, contributing to the dopaminergic cell death [111]. This synergistic outcome may represent a crucial phenomenon for the persistency of glial activation in Parkinsonism and could help to find specific therapeutic anti-inflammatory drugs to target cytokines to avert inflammation (Figure 4) [88]. Importantly, the role of TNF-*α* and IFN-*γ* should be further evaluated, especially in *postmortem* samples of Parkinsonian donors to take additional steps toward therapeutic strategies for PD.

### 4.4. Contribution of T Cells in the Proinflammatory Environment in Parkinsonism

The fact that cytokines and chemokines are able to recruit immune cells from the blood stream into the brain parenchyma has led to investigate the state of lymphocytes in Parkinsonian patients and in experimental models of PD (Figure 1). Early studies in Parkinsonian subjects have shown alterations of different T-cell subpopulations. An increase of gamma delta T cells, CD45RO^+^ memory T cells, and CD4^+^ T has been described in peripheral blood [115] and CSF of PD patients [116]. However, the function of these T-cell subsets in dopaminergic degeneration remains uncertain. Recent studies performed in *postmortem* brains of PD patients have described that CD4^+^ T cells specifically infiltrate in the dopaminergic degenerating areas [29]. Importantly, the putative mechanism of this subset of infiltrated T cells has been investigated in experimental models of PD. Parkinsonian rats and mice show an increase of CD4^+^ and CD8^+^ T cells after the neurotoxic insult [30], and similar to PD patients, active CD4 T cells are the subpopulation of lymphocytes critically increased peripherally [117, 118] and in the brain [29, 119]. Importantly, the infiltration of CD4 T cells seems to contribute to the dopaminergic neuro-degeneration, as it has been demonstrated in KO mice [29]. However, their particular involvement and mechanisms are still not clarified. Theoretically, CD4 T cells search for antigens displayed on MHC-II complexes (HLA-DR in humans) in degenerating areas, which are probably displayed by macrophages or microglial cells, but the antigen that might be presented by these MHC-II molecules is still conjectural. Some reports have suggested that *α*-synuclein, or different structural modifications of misfolded *α*-synuclein, may be a plausible antigen to be presented, which would be consistent with the hypothesis of an autoimmune disease, attacking certain forms of the protein accumulated by dopaminergic neurons [119]. This concept would involve the phagocytosis of certain dopaminergic neurons by microglia/macrophages, the digestion and processing of the protein, and the final presentation of the antigen in the microglial membrane, which remains to be elucidated. It is thought that Th17 response, a helper T-cell response involved in many autoimmune diseases, may be the adaptive immune response able to guide this autoimmune-like reaction against dopaminergic neurons. In fact, the immunization with nitrated *α*-synuclein exacerbates the dopaminergic degeneration in MPTP models in mice, which would support this hypothesis [120]. On the other hand, not every immunization and infiltrated T-cell population have the same effect on neurons. Studies report that immunizations that stimulate the proliferation and infiltration of certain T cells in the SNpc could be protective for dopaminergic neurons in MPTP-induced mice. Copolymer 1-immunized mice show CD4 T-cell infiltration in degenerating areas combined with IL-10 increase, which indicates a regulatory compound of T cells in contrast with the inflammatory Th17 autoimmune domain of T cells that may contribute to degeneration in other scenarios [121, 122]. The balance in the amount of circulating cytokines, IL-17 in Th17 response or IL-10 in regulatory responses, may lead to the differentiation of specific lymphocyte populations defining the degenerative outcome of the immune response.

Furthermore, changes in particular receptors on T-cell surface may also contribute to the differentiation of lymphocyte response. It has been described that T lymphocytes in PD patients show changes in their cytokines receptor binding. Particularly, IFN-*γ* receptors are decreased in T cells of PD patients, while TNF-*α* receptors are increased [123, 124]. This suggests that the signaling of circulating cytokines such as IFN-*γ* and TNF-*α* may also alter the polarization towards regulatory or inflammatory subsets modifying the outcome of the immune response. The study of the function of T cells in Parkinsonism represents a very promising field of research for the upcoming years.

## 5. Putative Disease-Modifying Anti-Inflammatory Drugs for PD

### 5.1. Nonsteroidal Anti-Inflammatory Drugs to Diminish Glial-Mediated Inflammation in Parkinson's Disease

Nonsteroidal anti-inflammatory drugs (NSAIDs) are one of the most reliable and promising therapies to avert the inflammatory response in PD. NSAIDs have successfully been tested in experimental models of Parkinsonism, reducing the brain inflammation and protecting dopaminergic neurons [125, 126]. This family of drugs includes compounds that inhibit the enzyme cyclooxygenase- (COX-) 1 and/or 2 [127]. Aspirin, for instance, inhibits both isoforms, while ibuprofen only inhibits COX-2 [127]. In the light of the clinical and experimental data, the use of COX-2 inhibitors seems to be the safest option for long-term treatments since COX-1 inhibition may cause damage of the gastric mucosa. Importantly, the epidemiological studies also point out to COX-2 inhibitors, as Ibuprofen, as the most effective anti-inflammatory drug reducing the risk of PD [4, 128]. Importantly, three independent meta-analyses have concluded that ibuprofen may have a protective effect in lowering the risk of PD [4, 128, 129]. The mechanism of action of COX-2 inhibitors in Parkinsonism seems to directly reduce the glial-mediated local inflammation in dopaminergic areas [130–133]. Particularly, the activation of microglia in the dopaminergic pathway in experimental Parkinsonism is mediated by COX-2 and contributes to neuronal death [134]. In fact, COX-2-deficient mice are protected against MPTP-induced dopaminergic degeneration and show reduced microglial inflammation, whereas mice lacking COX-1 present similar neuronal loss than wild type animals [130, 135]. Altogether, considering the safety and effectiveness, these data suggest that COX-2 inhibitors may be the drugs of choice for the treatment of PD [133, 136]. 

Regarding other anti-inflammatory drugs, new approaches have been tested that have successfully diminished some aspects of microglial activation. ROCK pathway has recently been described to play essential roles in microglial activation and dopaminergic neuron survival [137, 138]. ROCK-mediated mechanisms are involved in important features of microglial activation such as the increase of cell body size, increase of branches, and, importantly, the motility of microglial cells towards damaged dopaminergic neurons (Figure 3) [42]. Blocking ROCK reduces some of the features of microglial activation and preserves neurons from elimination [42]. Importantly, HA-1077, a potent ROCK inhibitor, commercially available as Fasudil, preserves neurons and fibers in MPTP models in mice and may represent a promising drug to be used for PD patients. In fact, Fasudil has the advantage that is currently used for patients with vasospasm in Japan, which indicates that Fasudil has already passed important steps regarding safety and toxicity controls for human consumption. Another approach that may be efficient in the near future involves targeting cytokines. Despite the clear involvement of certain cytokines in Parkinsonism, such as TNF-*α* and IFN-*γ*, the possibility of treating patients with cytokine inhibitors is still underdeveloped. There are a number of TNF-*α* and IFN-*γ* inhibitors designed with therapeutic purposes for other immune-related diseases. Artificially generated antibodies have been used successfully for autoimmune diseases such as multiple sclerosis and Crohn disease [107]. However, to predict the benefits for Parkinsonian patients may not be clear enough, and the possible side effects may be still too high to establish a solid therapeutic program. Furthermore, inhibiting fundamental cytokines such as TNF-*α* and IFN-*γ* may also weaken unnecessarily the immune system of the patients, which in elderly people may result in serious complications. Thus, new therapies for PD have to demonstrate a clear benefit/risk ratio to get into the market and the clinical practice.

### 5.2. Glucocorticoids as a Potential Therapy to Diminish Glial-Mediated Inflammation in Parkinson's Disease

Glucocorticoids (GC) are potent anti-inflammatory drugs generally used as effective treatment for many pathological conditions and immune-related diseases. As inflammation has become an important factor in PD pathogenesis, new studies have recently been published regarding the implication of GC in Parkinsonism [139, 140]. The inflammatory response, involving the release of cytokines into the blood circulation, has an effect on other anti-inflammatory systems such as the hypothalamic adrenal axis. Thus, the input of cytokines in the hypothalamus stimulates the release of ACTH that activates the production of glucocorticoids and mineralocorticoids from the adrenal gland, which may affect dopaminergic degeneration [139]. In fact, Parkinsonian patients show alterations in the adrenal axis, which in some cases are reflected in the increase of cortisol levels [93, 140]. In acute experimental models of PD, cortisol levels appear elevated weeks after the neurotoxic dopaminergic insult [141]. In contrast, in chronic Parkinsonian animals, no detectable modification of cortisol release is seen years after the induction of the dopaminergic degeneration [142]. These variations may reflect differences in the level of affectation of the hypothalamic pituitary axis between acute and chronic models of Parkinsonism. However, the measurement of cortisol levels is somehow controversial since the circulating amounts are susceptible to change with many different variables, such as external stressors, L-DOPA treatment, blood extraction schedule, and circadian rhythms [142, 143]. Although the accuracy of systemic measurements of circulating GC could be technically questioned, it is reasonable to think that the increase of GC may have physiological effects in the local inflammatory environment in Parkinsonian subjects' brains and consequently on dopaminergic neurons. Since GC receptor (GR) is ubiquitous, the presence of circulating GC may signal specific responses at different levels in the brain, affecting microglia, astroglia, and neurons. It has recently been reported that the activation of the microglial GR has a crucial effect in diminishing microglial cell activation and reduces dopaminergic degeneration in experimental Parkinsonism [140, 144]. 

Although GC may be effective in diminishing inflammation and could be considered as a promising option for PD treatment, there are many variables that are still unmanageable, together with the putative side effects that long-term GC treatments may provoke [145]. Key elements for the future research in this field would be the determination of effective doses with no deleterious effects and the development of GC able to target cell-specific GR and particular anatomical locations.

## 6. Concluding Remarks

Considering the latest experimental data, the epidemiological studies, and genetic analysis, inflammation is yet considered as an important contributor to dopaminergic degeneration in Parkinsonism. The local activation of glial cells, together with the chronic release of cytokines, and the putative role of infiltrated players, such as T cells, indicate that inflammatory response and immunity may be underling PD and may have important consequences for dopaminergic neurons. 

Among the common anti-inflammatory treatments commercially available, NSAIDs and GC are the putative choices. NSAIDs have been proven effective in preventing dopaminergic degeneration and reducing the proinflammatory response in experimental Parkinsonism. In the same way, GC such as dexamethasone or corticosterone provides similar results. Since the long-term treatment with GC may cause unsuitable side effects, COX-2 inhibitors, such as ibuprofen, seem to be the most effective and safe anti-inflammatory treatment, especially since it is supported by large-scale studies and meta-analyses.

In conclusion, there are increasing lines of evidence that anti-inflammatory drugs may be a beneficial treatment for PD. Most importantly, these treatments may represent a disease-modifying therapy in contrast with the current therapies that only treat symptoms, as L-DOPA and other dopaminergic agonists. However, it is still crucial to fully evaluate the putative side effects and the safety of long-term anti-inflammatory treatments, together with the study of the evolution of the inflammatory response of the patients to program new clinical trials with anti-inflammatory drugs for PD in the near future.
